# Nature’s Lab for Derivatization: New and Revised Structures of a Variety of Streptophenazines Produced by a Sponge-Derived *Streptomyces* Strain

**DOI:** 10.3390/md12041699

**Published:** 2014-03-25

**Authors:** Anna Lena Kunz, Antje Labes, Jutta Wiese, Torsten Bruhn, Gerhard Bringmann, Johannes F. Imhoff

**Affiliations:** 1 Kiel Center for Marine Natural Products at the Helmholtz Center for Ocean Research (GEOMAR), Am Kiel-Kanal 44, Kiel 24106, Germany; E-Mails: akunz@geomar.de (A.L.K.); alabes@geomar.de (A.L.); jwiese@geomar.de (J.W.); 2 Institute of Organic Chemistry, University of Wuerzburg, Am Hubland, Wuerzburg 97074, Germany; E-Mails: torsten.bruhn@uni-wuerzburg.de (T.B.); bringman@chemie.uni-wuerzburg.de (G.B.)

**Keywords:** marine natural product, phenazine, Baltic Sea, *Streptomyces*, *Halichondria panicea*, HPLC-DAD/MS, fermentation, PDE 4, antibiotic

## Abstract

Eight streptophenazines (A–H) have been identified so far as products of *Streptomyces* strain HB202, which was isolated from the sponge *Halichondria panicea* from the Baltic Sea. The variation of bioactivities based on small structural changes initiated further studies on new derivatives. Three new streptophenazines (I–K) were identified after fermentation in the present study. In addition, revised molecular structures of streptophenazines C, D, F and H are proposed. Streptophenazines G and K exhibited moderate antibacterial activity against the facultative pathogenic bacterium *Staphylococcus epidermidis* and against *Bacillus subtilis*. All tested compounds (streptophenazines G, I–K) also showed moderate activities against PDE 4B.

## 1. Introduction

**Figure 1:**
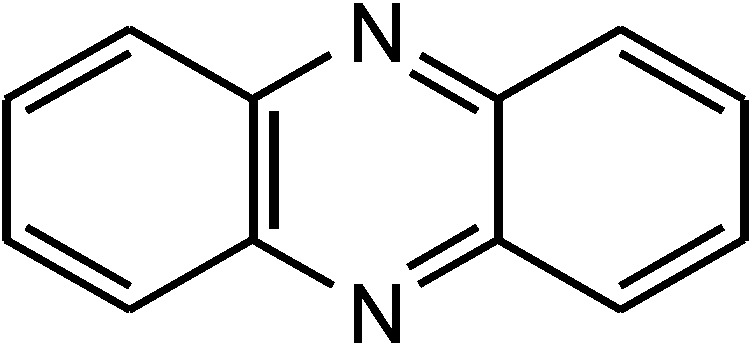
Core structure of phenazines.

Phenazines consist of two benzene rings linked through two nitrogen atoms and have been isolated as secondary metabolites of bacteria (Figure 1). Pyocyanin is the first natural phenazine discovered. It was found in the 1860s [1], when French clinicians observed blue colorations of pus-filled wounds of infected patients [2]. In 1882, Carle Gessard recognized bacteria as the producing organisms, which were named *Bacillus pyocyaneus* and are now known as *Pseudomonas aeruginosa* [1,2]. More than 6000 phenazines have been identified so far [3], with less than 170 of biological origin [4]. The larger part of phenazines from biological origin was isolated from terrestrial microorganisms, only a small number originates from marine habitats.

Due to the numerous possibilities of derivatization at the heteroaromatic chromophore, phenazines feature a large pool of structural diversity which covers a wide range of pharmaceutically relevant bioactivities with antibacterial, antifungal, antiviral and cytotoxic activities [3]. Minor differences in structure can have major effects on biological activity. The riminophenazine Clofazimine is a prominent example. It is used for the treatment of leprosy and was found to be potential for the treatment of drug-susceptible and drug-resistant *M*. *tuberculosis* strains [5]. Current studies of Liu *et al*. showed that the tricyclic phenazine skeleton is necessary for the activity against *M*. *tuberculosis*. Through replacement of the two phenyl groups at position C-2 and N-5, physicochemical properties as well as anti-tuberculosis potency could be enhanced [6].

This highlights the attractiveness to search for new phenazines and their derivatives and to analyze these in respect to their biological activities. Derivatives may be obtained by organic synthesis or by screening of biological phenazine producers. Among bacteria, a large number of phenazine derivatives can be found in a single strain indicating great efficiency of biological production. In former studies, this was shown for the marine *Streptomyces* strain HB202 aside from cultivation-based approaches also by genetic analysis [7,8]. Via detection of gene fragments *phz*E [7] and *phz*F [9], it was demonstrated that streptophenazines in this strain are produced by the regular phenazine biosynthesis pathway [1]. Eight derivatives of a new phenazine subgroup, the streptophenazines A–H, were isolated from cultures of this *Streptomyces* HB202 [10]. In part, the structures were revised later [11,12]. These streptophenazines have minor structural differences at positions C-1 and CH-2′, but exhibit important differences in their bioactivities. They are active against the Gram-positive bacteria *Bacillus subtilis* and *Staphylococcus lentus*.

Preliminary analysis of cultures from fermentation of *Streptomyces* sp. HB202 indicated the presence of a much larger number of derivatives as described before, exhibiting different physicochemical properties. Here, we characterize three new derivatives of the streptophenazines with respect to chemical structure and bioactivity. We also discuss the structural diversity gained by biotechnological approaches.

## 2. Results and Discussion

### 2.1. Metabolite Spectrum of HB202

**Figure 2:**
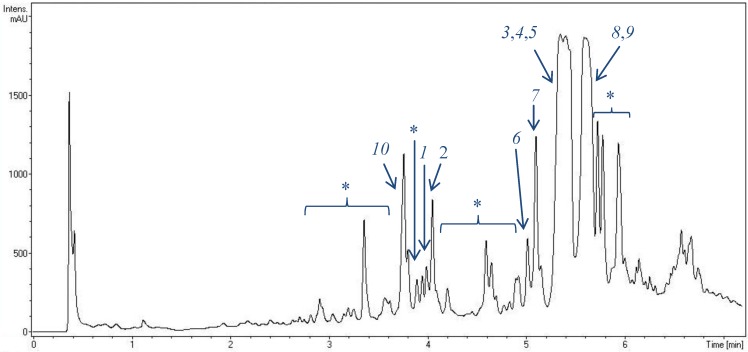
UV chromatogram of an extract of *Streptomyces* strain HB202 (at 250 nm) after fermentation in GYM4 medium for three days and extraction with EtOAc. For analysis, a RP-C18 column was used applying an H_2_O/CH_3_CN gradient on a VWR Hitachi Elite LaChrom system. Peak detection was done by DAD/MS. Numbers indicate identified streptophenazines. **1**, **2**, **3**: new streptophenazines (I–K), **4**: streptophenazine A, **5**: streptophenazine B, **6**: streptophenazine C, **7**: streptophenazine D, **8**: streptophenazine F, **9**: streptophenazine G, **10**: streptophenazine H, *: unknown streptophenazines (>10 different molecules).

Structures of eight streptophenazines, produced by *Streptomyces* strain HB202, were described previously [10,11,12]. Figure 2 demonstrates the presence of almost 20 phenazines in extracts of this strain after fermentation in GYM4 medium. They have at least small differences in polarity resulting in different retention times after separation on a non-polar C18 phase. Though some of these compounds are produced in decent quantities, others are present only in minor amounts in the extracts.

### 2.2. Structural Constitution of Streptophenazines

#### 2.2.1. Isolation and Structure of New Streptophenazines

The oily brown extract obtained from combined cultures was analyzed via HPLC-DAD/MS. Due to the characteristic UV maxima and the differences in polarity of the derivatives, phenazines are easily identified in the chromatogram and can be separated into pure compounds using a preparative HPLC-UV system. The structure elucidation of the three compounds was based on the interpretation of different NMR spectra (^1^H, ^13^C, DEPT, ^1^H–^13^C HSQC, ^1^H–^13^C HMBC, ^1^H–^1^H COSY) and HRESI-MS analysis.

**Figure 3:**
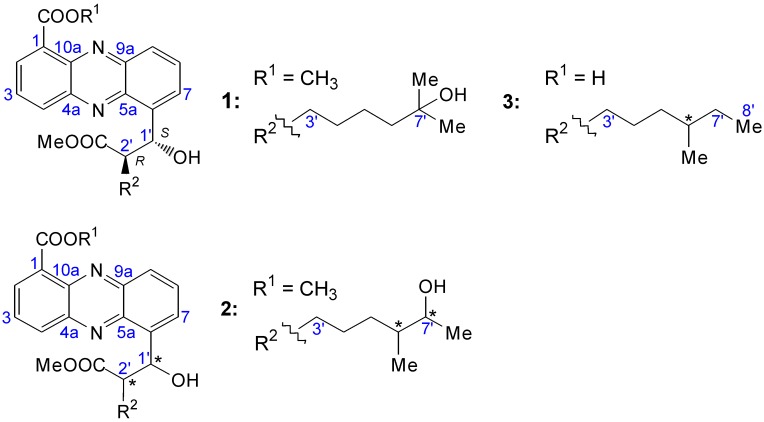
Structures of streptophenazines I–K (**1**–**3**).

Regarding the ^1^H-NMR spectra of the new compounds, it was obvious that all signals of protons belonging to the phenazine skeleton were identical. Significant differences could only be observed between the carbomethoxyl group and the carboxyl function at position C-1, as well as in the alkyl chain located at position CH-2′. Structures are shown in Figure 3.

Streptophenazine I (**1**) was isolated as a yellow solid. The characteristic UV spectrum (λ_max_ 215, 251, 347 (sh), 360 (sh), 366) in addition to the chemical shifts of the four quaternary carbons (δ 144.7, 142.9, 142.8, 141.9) adjacent to the two nitrogen atoms and the existence of aromatic hydrogen signals (δ 8.46–7.95) reasoned that **1** could be a phenazine. Through the alignment of NMR data (Table 1) with HRESI-MS analysis (*m/z* 455.21605 [M + H]^+^, calcd for C_25_H_31_N_2_O_6_: 455.21766), a molecular formula of C_25_H_31_N_2_O_6_ was established. The spectroscopic data indicated either a 1,6- or a 1,9-disubstituted phenazine skeleton. Due to this fact, data were compared to those of Yang *et al*. [11,12], who clearly defined the core structure of streptophenazines A, B, E and G to be 1,6-disubstituted via total synthesis as well as X-ray crystallographic data (streptophenazine A). Based on the high similarity of the NMR data in addition to the assumption of a common biosynthesic pathway of the derivatives in the same *Streptomyces* strain HB202, 1,6-disubstitution of new streptophenazines could be determined. The 1,6-disbubstitution was supplementary demonstrated by the use of NOESY spectra in the studies of Mitova *et al*. [10]. A singlet and the chemical hydrogen shift of 1-COOCH_3_ (δ 4.08), the corresponding carbon signal of the methyl group (δ 53.3) as well as the adjacent carbonyl signal (δ 168.9) indicated the presence of a methyl ester. The position was based on the ^1^H–^13^C HMBC coupling of the aromatic proton H-2 (δ 8.29). Furthermore the ^1^H–^13^C HMBC experiments revealed the occurrence of a second residue to be linked to the phenazine skeleton, an oxymethine substituent which led to a lowfield shift of CH-1′ (δ 71.4). The ^1^H–^1^H COSY correlation pointed out the presence of an adjacent methine proton (H-2′) whose carbon was bearing a carbomethoxy group. The comparison of ^1^H–^13^C HMBC with the DEPT spectrum led to the following methylene protons H-3′-H-6′ as well as the oxymethine substituent (CH-7′) which terminated the alkyl chain in addition to the two methyl residues CH_3_-8′ and 7′-CH_3_.

**Table 1:** NMR spectroscopic data of **1**.

Position	δ_C_, DEPT	δ_H_, *J* [Hz]	COSY	HMBC
1	132.8, C			
2	133.6, CH	8.29 dd (6.9, 1.4)	3	1-COOCH_3_, 10a, 4
3	130.9, CH	7.96 m	2, 4	4a, 1
4	134.8, CH	8.46 dd (8.8, 1.4)	3	10a, 2
4a	144.7, C			
5a	142.9, C			
6	143.2, C			
7	130.2, CH	8.03 m	8	1′, 5a, 9
8	132.5, CH	7.99 m	7, 9	6
9	130.4, CH	8.26 dd (8.6, 1.5)	8	7, 5a
9a	142.8, C			
10a	141.9, C			
1′	71.4, CH	6.16 d (7.7)	2′	2′-COOCH_3_, 5a, 7
2′	55.0, CH	3.27 ddd (10.1, 7.7, 4.3)	1′	
3′	30.5, CH_2_	1.26 m, 1.74 m		
4′	25.2, CH_2_	1.17 m		
5′	29.2, CH_2_	1.17 m		
6′	44.6, CH_2_	1.24 m		4′
7′	71.3, C			
8′	29.2, CH_3_	1.03 s		5′, 6′
7′-CH_3_	29.2, CH_3_	1.03 s		5′, 6′
1-COOCH_3_	168.9, C			
1-COOCH_3_	53.3, CH_3_	4.08 s		1-COOCH_3_
2′-COOCH_3_	176.8, C			
2′-COOCH_3_	52.1, CH_3_	3.63 s		2′-COOCH_3_

**Figure 4:**
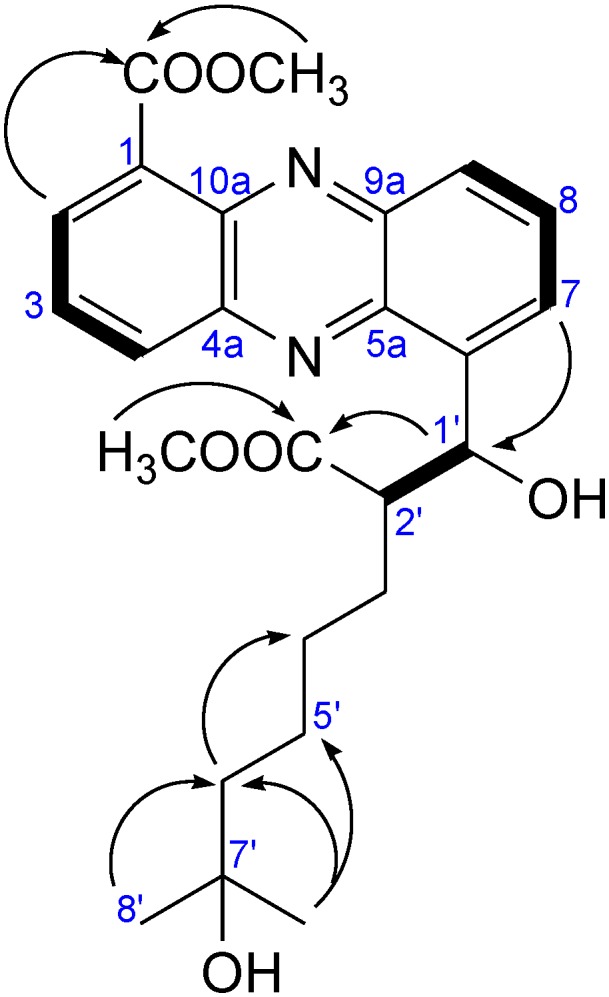
Selected ^1^H–^13^C HMBC (arrows) and ^1^H–^1^H COSY (bold) correlations, relevant for structure elucidation of **1**.

The structure of **1** was quite similar to that of streptophenazine H (**10**) with the only difference of one missing methylene at **10**, resulting in a molecular weight with 14 mass units less. The structure of **1** is shown in Figure 3, as well as in Figure 4 illustrated through arrows of ^1^H–^13^C HMBC correlations. Spectroscopic data are shown in Table 1.

Streptophenazine J (**2**) is an isomer of **1** comparing the molecular formula C_25_H_31_N_2_O_6_, established via HRESI-MS (*m/z* 455.21637 [M + H]^+^, calcd for C_25_H_31_N_2_O_6_: 455.21766), the UV spectrum and the NMR signals of the skeleton. Analysis of ^1^H–^1^H COSY data showed that one of the methyl groups (7′-CH_3_ of **1**) is missing. Instead, a ^1^H–^1^H COSY correlation of H-6′ pointed out the presence of another methyl group at CH-6′ in this molecule. Unfortunately, **2** is stereochemically unstable for which reason it is difficult to do an accurate structure elucidation. This information led to the structure of **2** as shown in Figure 3 with undefined absolute configuration.

Streptophenazine K (**3**) showed structural similarities to streptophenazine G (**9**) except an unesterified carboxyl group at position C-1. This was based on the highfield shift of C-1 (δ 126.6) as well as on the observations of the ^1^H-NMR spectrum similar to that of **9** with the appearance of only one methoxyl group (2′-COOMe) and a difference of 14 mass units. HRESI-MS pointed out the molecular formula C_24_H_29_N_2_O_5_ (*m/z* 425.20875 [M + H]^+^, calcd for C_24_H_29_N_2_O_5_: 425.20710). The structure of **3** is shown in Figure 3.

The relative configurations of streptophenazines I-K **1**–**3** at C-1′ and C-2′ were determined using the ^3^*J* coupling constants between the protons at these carbon atoms. Yang *et al*. [11,12] found that the *like*-configuration (*R*,*R* or *S*,*S*) in the very similar streptophenazines A or G had a coupling constant of 6.5 Hz, while the *unlike*-configuration (*R*,*S* or the *S*,*R*) had one of 7.5 Hz. The compounds **1**, **2** and **3** showed coupling constants of 7.7, 7.8 and 7.5 Hz, respectively, and thus should all have the *unlike*-configuration at C-1′ and C-2′.

**Figure 5:**
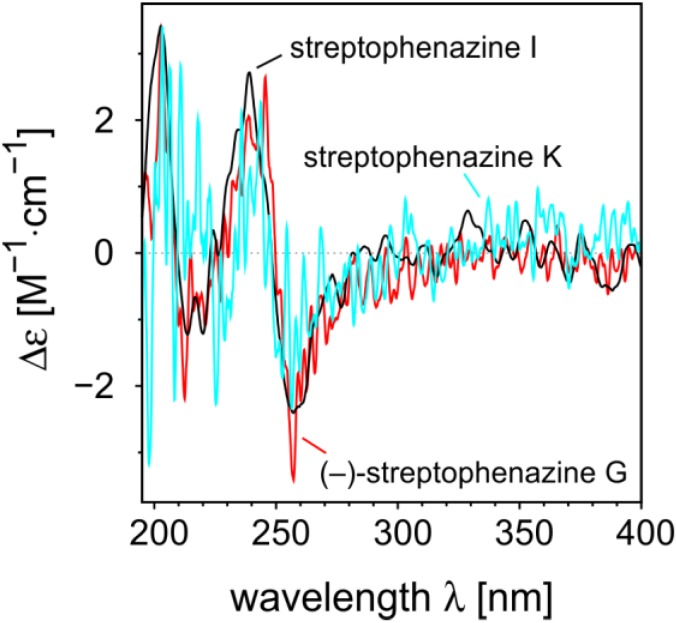
Comparison of the CD spectra of (–)-streptophenazine G, streptophenazine I, and streptophenazine K.

For assignment of the absolute configuration, CD measurements were performed of streptophenazines I and K, and of (–)-streptophenazine G **9** whose absolute configuration was known from total synthesis [12]. Despite the presence of a large chromophore, all compounds surprisingly gave CD curves of very low intensity (Figure 5). This was explained by the fact that structures of this type have pseudo-enantiomeric conformers, in which the stereocenters in the side chain are on different sides of the chromophore. These conformations lead to nearly mirror-image like single CD spectra, and thus in the overall CD curve the net rotational strengths nearly cancel out. However, it was possible to get reproducible spectra and we were able to elucidate the absolute configurations of the compounds by comparing the experimental CD spectra of **1** and **3** with that of (–)-streptophenazine G **9**. They have the same chromophore and the free carboxylic acid group of streptophenazine K will influence the CD curve in comparison to the ester group of streptophenazines I and G only marginable. Of course, the comparison of the CD spectra can only give the absolute configuration of C-1′ (that of C-2′ is then deduced from the coupling constants as described above). All other stereocenters (C-6′ in (–)-streptophenazine G and in streptophenazine K) are too far away from the chromophore to have an impact on the electronic CD. Finally, the nearly identical CD spectra of streptophenazines I and K with that of (–)-streptophenazine G proved that they were all 1′*S*,2′*R*-configured as shown in Figure 3. Unfortunately, it was not possible to measure a reproducible CD spectrum of streptophenazine J **2**, most probably due to the generally low intensity of CD curves of the spreptophenazines in combination with the fact that it was stereochemically not stable.

#### 2.2.2. Structure Revision of Streptophenazines B–F and H and Structural Comparison

The structures of streptophenazines A (**4**), B (**5**), E (**11**) and G **9** have recently been revised [11,12]. The revision of **5** and **11** is shown in the supplement of Yang *et al*. [11]. Due to new interpretations of NMR spectra, which indicate that current structures of these derivatives are not correct [10], a revision is also needed for the streptophenazines C (**6**), D (**7**), F (**8**) and H **10**.

**Figure 6:**
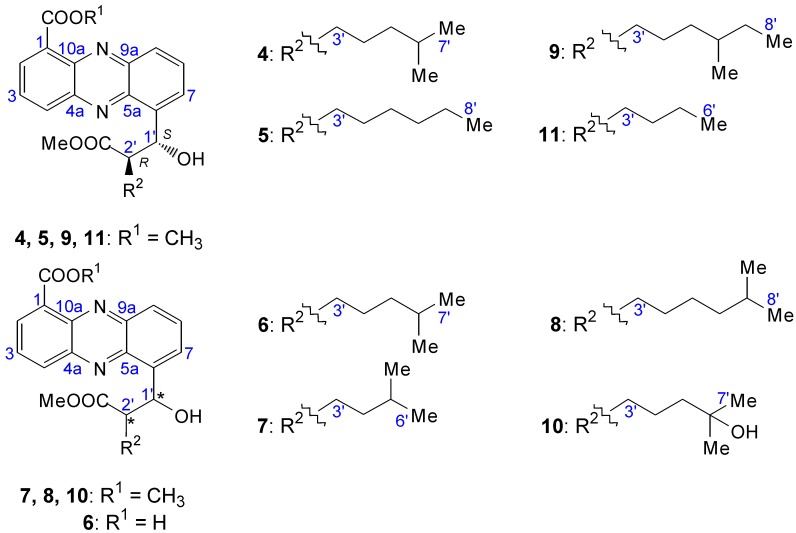
Revised structures of streptophenazines A–H [10,11,12].

The result of the new investigation was, that the published structures of Mitova *et al*. [10] are incorrect, and consequential the residues at CH-1′ and CH-2′ have to be switched. This was shown by the ^1^H–^13^C HMBC correlation of H-7 to CH-1′ and in addition to that the lowfield shift of CH-1′ in the ^13^C and ^1^H spectra, which was caused by the adjacent hydroxyl group. The revised structures are shown in Figure 6. 

### 2.3. Bioactivity Assays

Bioactivities of **1**–**3** were measured because of their novelty and compared with those of **9** as a known representative of streptophenazines. The results are shown in Table 2.

**Table 2:** Bioactivities of compounds **1**–**3** and **9**.

	*Bacillus subtilis* (IC_50_ [µM])	*Staphylococcus epidermidis* (IC_50_ [µM])	PDE 4B (IC_50_ [µM])
Streptophenazine G **9**	8.2 µM (± 0.9)	8.4 µM (± 0.5)	5.2 (± 1.0)
Streptophenazine I **1**	not active	not active	11. 6 (± 1.1)
Streptophenazine J **2**	not active	not active	12.0 (± 0.9)
Streptophenazine K **3**	21.6 (± 6.8)	14.5 µM (± 2.0)	12.2 (± 2.0)
Rolipram	not determined	not determined	0.75 (± 0.05)

Streptophenazines A–H have been shown to be antibiotically active against the Gram-positive bacteria *Bacillus subtilis* and *Staphylococcus lentus* [10]. Because of its clinical relevance, *Staphylococcus epidermidis* was used as a test strain in this study in addition to *Bacillus subtilis* [13]. **3** and **9** inhibited the growth of *Staphylococcus epidermidis* with IC_50_ values of 14.5 µM and 8.4 µM, respectively. Both **3** and **9** were also active against *Bacillus subtilis* with IC_50_ values of 21.6 µM and 8.2 µM, respectively. No antibacterial activity was observed for the compounds **1** and **2**. All four compounds **1**–**3** and **9** showed inhibitory activity against the enzyme phosphodiesterase (PDE 4B).

There is a great interest in the discovery of new drugs for the treatment of inflammatory diseases such as COPD. Since it was observed, that the function of inflammatory cells could be inhibited by an increase of 3′5′-cyclic adenosine monophosphate (cAMP), inhibitors of the phosphodiesterases (PDEs) were considered as promising drug candidates [14]. The increase in the cAMP results by the inhibition of hydrolyses of cAMP to the inactive 5′ monophosphate (5′-AMP). As a result, there is a reduction in the inflammatory cell activity, an inhibition of fibrosis and a relaxation of smooth muscles [14]. The non-selective PDE inhibitor theophylline (since the 1930s) and the selective PDE4 inhibitor benzamide roflumilast-*N*-oxide, which has been approved by the FDA in 2011, are drugs for the treatment of COPD [15]. Roflumilast-*N*-oxide showed IC_50_ values in the range of 0.4 to 7.8 nM dependent on the used PDE4 isoenzyme [16]. Further PDE4 inhibitors, such as rolipram, cilomilast, oglemilast, tetomilast, ONO-6126, and ELB353 are in several stages of clinical trials. As far as known, phenazines were not yet identified as PDE4 inhibitors. In this study, moderate activity against PDE4 was observed for all four streptophenazines tested.

### 2.4. Biotechnological Upscaling

Lead structure development and entering of (pre-)clinical phases for newly identified natural products depend on sufficient supply of material. For this purpose, chemical synthesis and biotechnological production have to be considered and compared in terms of feasibility, effort and costs. When aiming for a variety of derivatives, biological production may have advantages, as it seems to be a common feature of many microbes to produce more than one derivate of a given compound. However, upscaling of the biotechnological production from in most cases Erlenmeyer flask cultures to controllable stirred tank reactors (STR) is a challenge, as the scaling itself is a change of cultivation conditions that may lead to a change of the secondary metabolite profile [17,18]. Especially in the case of HB202 being very sensitive to changes in the cultures, the scaling did bear many risks.

**Figure 7:**
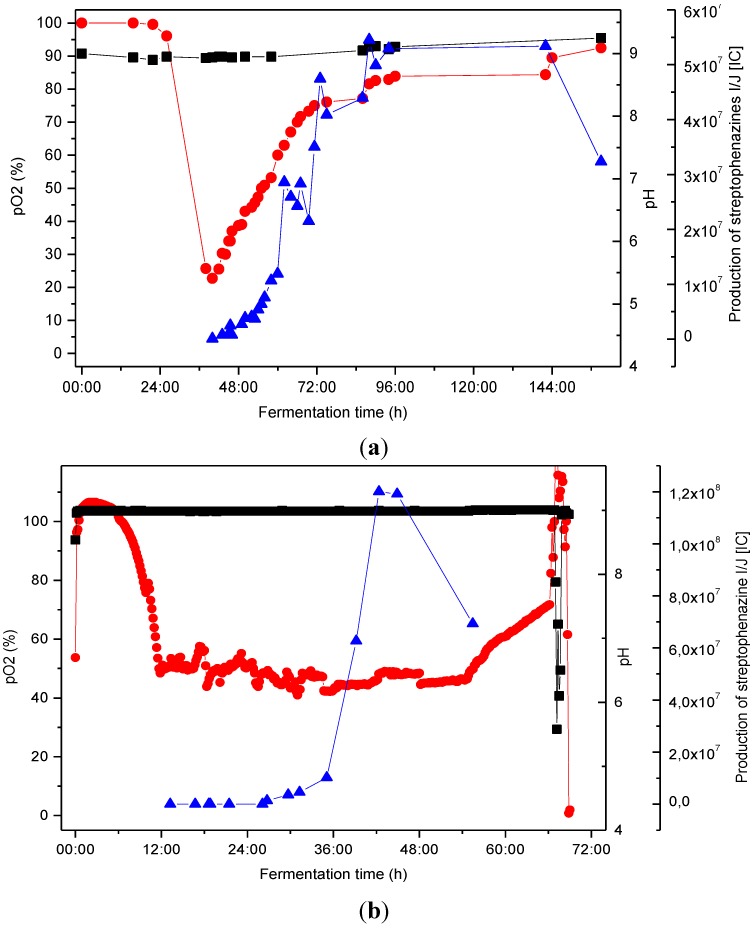
Fermentation of *Streptomyces* HB202 at 28 °C in GYM medium. Comparison of streptophenazine production in consideration of pO_2_ and pH within two different tank reactors using stable pH conditions at pH 9; (**a**) 10 L culture in a stirred tank reactor (STR) (**b**) 200 L culture in a STR; 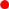 pO_2_ [%], ■ pH, 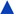 production of streptophenazines I/J [IC].

Figure 7 illustrates the scaling process, which included optimization of cultivation conditions with the aim of enhancing production of the streptophenazines at each step. The original cultures that lead to the identification of the streptophenazines were done in Erlenmeyer (EM) flasks. EM flasks were used for cultivation in 1 L scale at different pH values, pH 9 turned out to be best for production of secondary metabolites by *Streptomyces* sp. HB202. For all subsequent experiments in STR, the pH value was set to constant pH 9 being a relatively harsh condition. Controlled STR cultures where done in 10 L and 200 L cultures. Figure 7 shows the process of production in the two fermenter systems.

Streptophenazine production occurred in the late exponential phase (as shown by the rising pO_2_). In the 10 L scale, the maximum yield was obtained after 90 h. The process time was decreased to 40 h by scaling to 200 L (maximum yield). Additionally, the maximum yield could further be increased within this step. A proceeding cultivation led to a decrease of streptophenazines. However, not only the streptophenazines I/J were found but a variety of further streptophenazine derivatives (minimum 20 different derivatives).

Exemplary, the yield was determined for the derivative pair streptophenazine I and J. The Erlenmeyer culture yielded 104 µg/L, the same order was obtained in the 10L STR (109 µg/L). The scaling to the 200 L culture doubled the yield to 196 µg/L.

**Figure 8:**
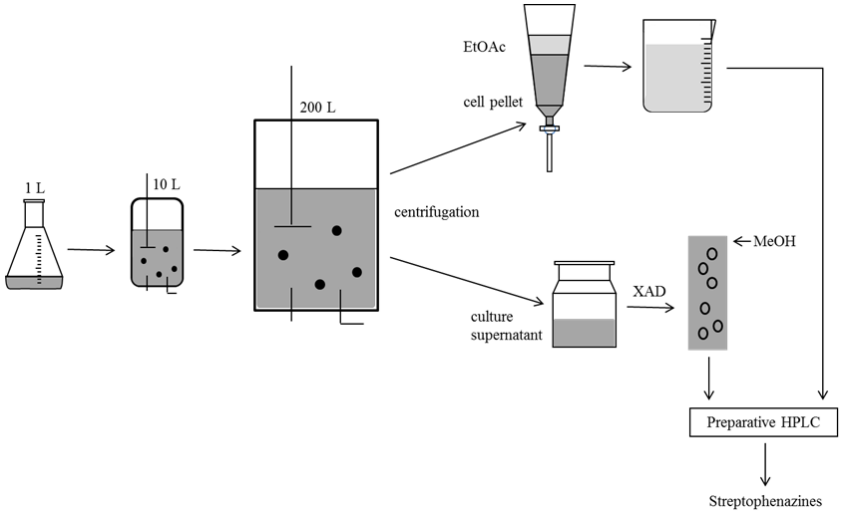
Process of sample treatment after cultivation procedure; Separation into culture, supernatant and cell pellet and subsequent extraction with EtOAC and partition of cell components; Assembling of both extracts and purification using preparative HPLC.

The scaling of the fermentation led to an increase in product yield and a significant reduction of process time, which was further reduced by an optimization of the capture step. The metabolites of HB202 were secreted into the culture medium as well as are stored in the cells. Hence, both had to be collected and processed subsequent to the fermentation. The standard method in small scale comprised solvent extraction with two volumes of EtOAc per liter of culture broth. To avoid the large amount of solvent necessary for the processing of 200 L of culture, a capture step was introduced. The scavenger XAD was used to adsorb small molecules from the broth (Figure 8). Downstream processing in marine biotechnology seems to be the neglected child of bioprocess engineering. The application of a scavenger is only the first step of optimization of the downstream process. To be able to valorize compounds from marine microorganisms, big efforts must be made in the downstream process [19].

Because of a production decline in the tank reactors at the end of fermentation and due to losses during the subsequent purification of the streptophenazines from the crude extract only 56%–57% of the compounds could be gained. 505.5 µg were purified in total from the 10 L STR (57% of the produced amount), 22.4 mg from the 200 L system (56%). The yields and losses may be compared to the yield of stereoselective synthesis, as shown by Yang *et al*. streptophenazine A (1′*S*,2′*R*) was synthesized with a yield of 60% in the last step (60 mg from 135 mg of the precursor methyl 6-((1*S*,2*R*)-2-((*S*)-4-benzyl-2-oxooxazolidine-3-carbonyl)-1-hydroxy-6-methylheptyl)phenazine-1-carboxylate). Organic synthesis was shown to be relatively effective for some streptophenazine derivatives. In terms of production costs and time, this synthesis is quite promising. However, biotechnological production, as shown in our experiments, can be feasible as well: A stirred tank reactor production followed by a XAD capture step and subsequent HPLC based purification yields appr. 20 mg of product. The biotechnological approach gains a natural “derivatization”, up to 20 different streptophenazines may be produced by one process. A good understanding of the production process will provide ample opportunities for successful scale up to the industrial level. Discovery scientists can have an impression on the various options available for scale-up of their lead product. A recent study demonstrated that in many cases of marine biotechnology, a suboptimal production yield was gained as technological barriers were limiting the process [20]. A special challenge to bioengineers is e.g., provided by barophilic strains originating from depths of, say, nearly 11,000 m, or from hydrothermal vents [19]. However, in the case of HB202, a standard optimization process could be applied.

## 3. Experimental Section

### 3.1. General Experimental Procedures

The optical rotation was measured on a Perkin-Elmer model 241 polarimeter.

NMR spectra were recorded on a Bruker DRX500 spectrometer (500 and 125 MHz for ^1^H and ^13^C NMR, respectively), using the signals of the residual solvent protons and the solvent carbons as internal references (δ_H_ 3.31 and δ_C_ 49.15 ppm for CH_3_OH-*d*_4_). High-resolution mass spectra were acquired on a benchtop time-of-flight spectrometer (MicrOTOF-II, Bruker Daltonics, Bremen, Germany) with positive electrospray ionization.

Analytical reversed phase HPLC-DAD/MS experiments were performed using a C18 column (Phenomenex Onyx Monolithic C18, 100 × 3.00 mm) applying an H_2_O (A)/CH_3_CN (B) gradient with 0.1% formic acid added to both solvents (gradient: 0 min 5% B, 4 min 60% B, 6 min 100% B; flow 2 mL/min) on a VWR Hitachi Elite LaChrom system (DAD-detector: Hitachi L-2450 diode array detector) coupled to an ESI-ion trap detector with positive ionization (Esquire 4000, Bruker Daltonics).

Semi-preparative HPLC-DAD was carried out using a Phenomenex normal phase column (Luna 5U Silica (2), 100A, 250 × 10.00 mm, 5 micron).

For preparative fractioning a HPLC-UV system (VWR International LaPrep, Pump P311, Detector P110, autosampler smartline 3900) with a RP-C18 column (Phenomenex Gemini C18, 110A, AXIA, 100 × 50.00 mm) applying an H_2_O (A)/CH_3_CN (B) gradient with 0.1% formic acid (gradient: 0 min 10% B, flow 40 mL/min; 0.5 min 10% B, 17.5 min 60% B, 22 min 100% B; flow 100 mL/min) was used.

### 3.2. Isolation and Identification of Strain *Streptomyces* sp. HB202

Strain HB202 was isolated from the marine breadcrumb sponge *Halichondria panicea* (class Demospongia) collected from the Baltic Sea (Germany) [8]. The taxonomic identification was based on morphological criteria such as the cream color, a compact-solid surface, a sheet with neat round pores (osculae) and anastomosing hollow branches with osculae at the tip [21]. Isolation and identification of strain HB202 was described by Mitova *et al*. [10]. The most closely related type strains according to the 16S rRNA gene sequence (GQ863918, 1473 bp) were *Streptomyces tanashiensis* IFO 12919^T^ (GenBank/EMBL/DDBJ acc. No. AY999856) and *Streptomyces griseoplanus* AS 4.1868^T^ (GenBank/EMBL/DDBJ acc. No. AY999894) with a sequence similarity of 99.93% for both strains. Several type strains of *Streptomyces* species exhibited sequence similarities ≥99.80%, such as *Streptomyces badius* NRRL B-2567 (GenBank/EMBL/DDBJ acc. no. AY999783), *Streptomyces mediolani* LMG 20093^T^ (GenBank/EMBL/DDBJ acc. No. AJ781354) and *Streptomyces griseus* ATCC 51928^T^ (GenBank/EMBL/DDBJ acc. No. AF112160).

### 3.3. Cultivation, Extraction and Substance Characterization

*Streptomyces* sp. HB202 was cultivated in 1 L Erlenmeyer flasks using different pH values. The cultivation was subsequently scaled up in stirred tank reactors in 10 L (Braun Biostat, glass tank, containing 8 L culture) and in 300 L scale (Infors Techfors 300, steel tank, containing 200 L culture) using pH 9 (controlled via addition of 3 M NaOH).

Precultures were established from cryocultures in a standardized manner as the strain was very susceptible for changes and reacted with a dramatic change of the metabolite spectrum. Precultures were carried out on agar plates, which were incubated after inoculation using the Microbank system (MAST DIAGNOSTIKA, Reinfeld, Germany) and in liquid medium at 28 °C in the dark.

In each case, the seed medium for the marine strain *Streptomyces* HB202 consisted of 4 g glucose, 4 g yeast extract, 4 g malt extract per 1 L demineralized water. Cultivation in Erlenmeyer flasks was done for up to seven days in different starting pH values. While the 10 L fermenter was run for 159 h at 28 °C, the pilot scale fermenter was run for 69 h. In the STR systems, pH, oxygen, CO_2_-outlet and stirring speed were controlled. The oxygen content in the medium was set to a minimum of 30% air saturation. Foam formation was stopped by addition of antifoam (Sigma, Taufkirchen, Germany).

After cultivation, cells were separated from the culture broth by means of centrifugation. For the 1 L and 10 L scale, culture supernatant and cells were extracted by addition of 2 Volumes EtOAc. The organic solvent was separated and concentrated to dryness under reduced pressure. For the 200 L scale, cells (appr. 6 L cell pellet) were treated in the same manner as cells from smaller cultivations. The culture supernatant was supplemented with XAD16 (Amberlite, 5 g/L). The XAD was recovered and transferred to a glass column. Substances were washed out using MeOH. Both crude extracts were assembled and subjected to preparative HPLC-UV. The concentrated fractions were subsequently purified using semi-preparative HPLC-DAD. The structures of the substances were elucidated using NMR and UV/VIS spectra.

Streptophenazine I **1**: Yellow solid; 3.0 mg, 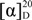 −21° (c 0.0475, MeOH); UV (MeOH) λ_max_ 215, 251, 347 (sh), 360 (sh), 366; for 1D and 2D NMR data (MeOH-*d*_4,_ 500 MHz and 125 MHz, respectively) see Table 1; HRESIMS *m/z* 455.21605 [M + H]^+^ (calcd for C_25_H_31_N_2_O_6_: 455.21766).

Streptophenazine J **2**: Yellow solid; 6.6 mg, 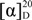 −18° (c 0.1125, MeOH); UV (MeOH) λ_max_ 215, 251, 348 (sh), 360 (sh), 366; ^1^H NMR (CH_3_OH-d_4,_ 500 MHz) δ_H_ 8.45 (1H, m, H-4), 8.28 (1H, dd, *J =* 7.0, 1.4, H-2), 8.26 (1H, dd, *J =* 8.6, 1.5, H-9), 8.03 (1H, d, *J =* 6.9, H-7), 7.99 (1H, m, H-8), 7.95 (1H, m, H-3), 6.17 (1H, d, *J =* 7.8, H-1′), 4.08 (3H, s, 1-COOCH_3_), 3.63 (3H, s, 2′-COOCH_3_), 3.44 (1H, m, H-7′), 3.27 (1H, ddd, *J =* 10.3, 7.8, 4.4, H-2′), 1.73 (1H, m, H-3′a), 1.29 (m, 1H, H-5′a), 1.25 (1H, m, H-3′b), 1.24 (2H, m, H-4′), 1.23 (1H, m, H-6′), 0.97 (3H, m, H-8′), 0.93 (1H, m, H-5′b), 0.73 (3H, m, 6′-CH_3_); ^13^C NMR (CH_3_OH-d_4,_ 500 MHz): δ_C_ 176.8 (C, 2′-COOCH_3_), 168.9 (C, 1-COOCH_3_), 144.7 (C, C-6), 143.1 (C, C-5a), 142.9 (C, C-4a), 142.8 (C, C-9a), 141.8 (C, C-10a), 134.8 (CH, C-4), 133.6 (CH, C-2), 132.7 (C, C-1), 132.5 (CH, C-8), 130.8 (CH, C-3), 130.5 (CH, C-9), 130.2 (CH, C-7), 72.0 (CH, C-7′), 71.3 (CH, C-1′), 55.0 (CH, C-2′), 53.3 (CH_3_, 1-COOCH_3_), 52.1 (CH_3_, 2′-COOCH_3_), 40.8 (CH, C-6′), 33.3 (CH_2_, C-5′), 30.8 (CH_2_, C-3′), 26.3 (CH_2_, C-4′), 20.2 (CH_3_, C-8′), 14.7 (CH_3_, 6′-CH_3_); HRESIMS *m/z* 455.21637 [M + H]^+ ^(calcd for C_25_H_31_N_2_O_6_: 455.21766).

Streptophenazine K **3**: Yellow solid; 10.0 mg, 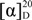 −64° (c 0.0900, MeOH); UV (MeOH) λ_max_ 216, 252, 271 (sh), 361 (sh), 370; ^1^H NMR (CH_3_OH-d_4,_ 500 MHz) δ_H_ 8.81 (1H, dd, *J =* 7.0, 1.4, H-2), 8.56 (1H, dd, *J =* 8.7, 1.4, H-4), 8.26 (1H, dd, *J =* 7.9, 2.2, H-9), 8.11 (1H, m, H-7), 8.09 (1H, m, H-8), 8.08 (1H, m, H-3), 6.18 (1H, d, *J =* 7.5, H-1′), 3.62 (3H, s, 2′-COOCH_3_), 3.25 (1H, ddd, *J =* 10.4, 7.5, 4.6, H-2′), 1.72 (1H, m, H-3′a), 1.30 (1H, m, H-7′a), 1.24 (2H, m, H-4′), 1.11-1.19 (3H, m, H-3′b, H-5′a, H-6′), 0.93-1.05 (2H, m, H-5′b, H-7′b), 0.75 (3H, m, 6′-CH_3_), 0.74 (3H, m, H-8′); ^13^C NMR (CH_3_OH-d_4,_ 500 MHz): δ_C_ 176.6 (C, 2′-COOCH_3_), 168.5 (C, 1-COOH), 143.7 (C, C-6), 143.6 (C, C-4a), 143.4 (C, C-5a), 141.6 (C, C-9a), 141.3 (C, C-10a), 138.1 (CH, C-2), 136.5 (CH, C-4), 134.4 (CH, C-8), 131.7 (CH, C-3), 130.6 (CH, C-7), 128.7 (CH, C-9), 126.6 (C, C-1), 70.9 (CH, C-1′), 54.9 (CH, C-2′), 52.1 (CH_3_, 2′-COOCH_3_), 37.4 (CH_2_, C-5′), 35.5 (CH, C-6′), 30.7 (CH_2_, C-3′), 30.4 (CH_2_, C-7′), 26.0 (CH_2_, C-4′), 19.5 (CH_3_, 6′-CH_3_), 11.8 (CH_3_, C-8′); HRESIMS *m/z* 425.20875 [M + H]^+ ^(calcd for C_24_H_29_N_2_O_5_: 425.20710).

^1^H NMR spectra, COSY spectra, ^13^C NMR spectra, DEPT spectra, HSQC spectra and HMBC spectra of compounds **1**–**3** are available as Supplementary Information.

### 3.4. Determination of Biological Activity

The antimicrobial assay was performed using *Staphylococcus epidermidis* DSM 20044 and *Bacillus subtilis* DSM 347. Overnight cultures of the strains cultivated in tryptic soy broth were diluted to an OD (600 nm) of 0.01–0.05. The assays were prepared by transferring 2 µL of a 10 mM solution of the tested compound dissolved in DMSO into one well of a 96-well microtiter plate containing 200 µL of cell suspension culture. After the incubation of the microtiter plates for 5 h at 37 °C, 10 µL of a resazurin solution (0.2 mg/mL phosphate-buffered saline) was added to each well and the plates were incubated again for 5–30 min. Cell viability was assessed by the reduction of resazurin to resorufin by measuring the absorbance 600 nm (reference 690 nm). The resulting values were compared with a positive control (10 mM chloramphenicol) and a negative control (no compound) on the same microtiter plate.

The inhibitory activity of the enzyme phosphodiesterase type 4B was determined according to Schulz *et al*. [22].

## 4. Conclusions

The chromatogram of the marine *Streptomyces* strain HB202 showed a number of peaks which indicate the appearance of a huge number of phenazines in one fermentation approach. Eight of these streptophenazines were described by Mitova *et al*. [10] and revised by Yang *et al*. (A, B, E [11], G [12]) and in this publication (C, D, F, H). The structure of three new streptophenazines was elucidated and their activity in selected bioassays tested in this work, revealing that all derivates showed moderate activities against PDE4B. Due to the fact that so far phenazines are not known as PDE inhibitors, these bioactivities indicate additional medical indications for the pharmaceutical use of phenazines. E.g. streptophenazines may be considered for the treatment of COPD.

Streptophenazines occur with an amazing variety of derivatives in nature, in this case with more than 20 derivatives produced by a single marine *Streptomyces* strain. This “biological derivatization” allows structure-function relationship studies without extensive chemical synthesis approaches. In terms of enhancing production levels for biotechnological production of streptophenazines, we demonstrated production at the 200 L scale in stirred tank reactors, yielding 39.3 mg/L streptophenazine I and J with a subsequent capture step and purification of the compounds via preparative HPLC.

## Supplementary Files

Supplementary File 1 Supplementary Information (PDF, 1632 KB)
