# Supplementary material for: Nature’s Lab for Derivatization: New and Revised Structures of a Variety of Streptophenazines Produced by a Sponge-Derived Streptomyces Strain
Source: Mar Drugs. 2014 Mar 25;12(4):1699–714. doi: 10.3390/md12041699 (PMC4012441; doi:10.3390/md12041699)
Supplement: Supplementary File 1 — Supplementary Information (PDF, 1632 KB) [file marinedrugs-12-01699-s001.pdf]

Supplementary Information

Figure S1. ¹H NMR of compound streptophenazine I (**1**) in MeOD-d_4.

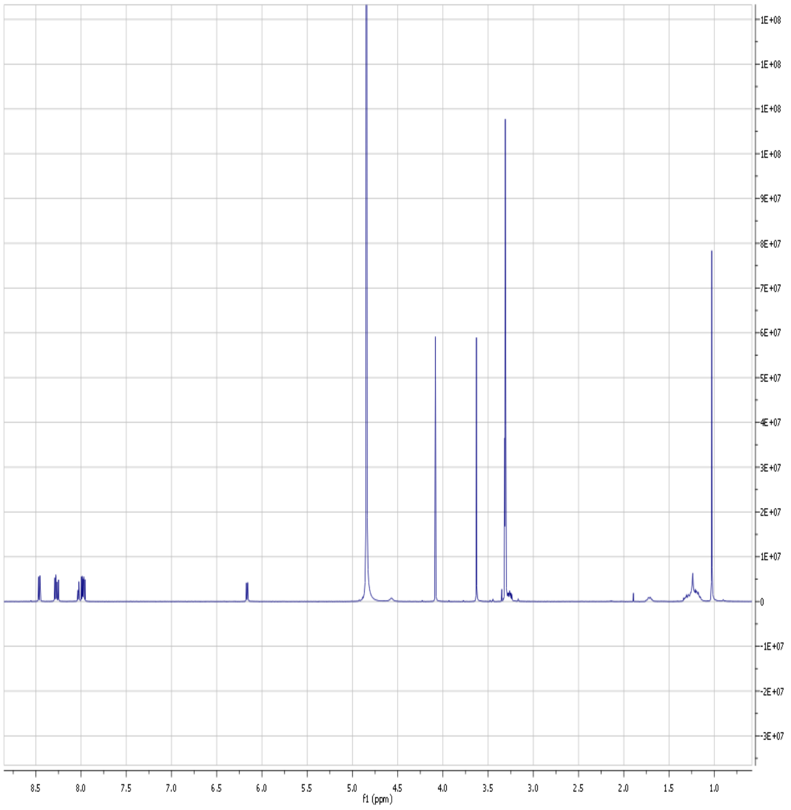

Figure S2. COSY of compound streptophenazine I (**1**) in MeOD-d_4.

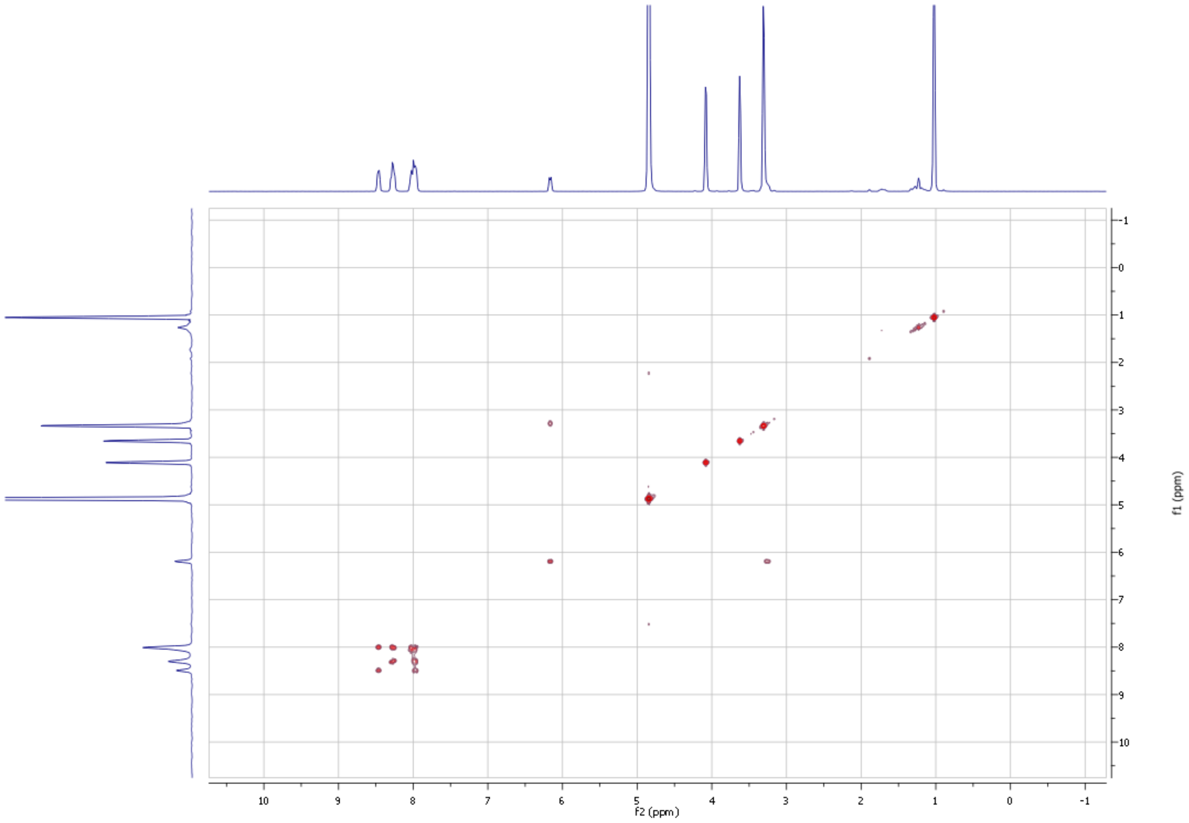

Figure S3. ^{13}C NMR of compound streptophenazine I (**1**) in MeOD-d_4.

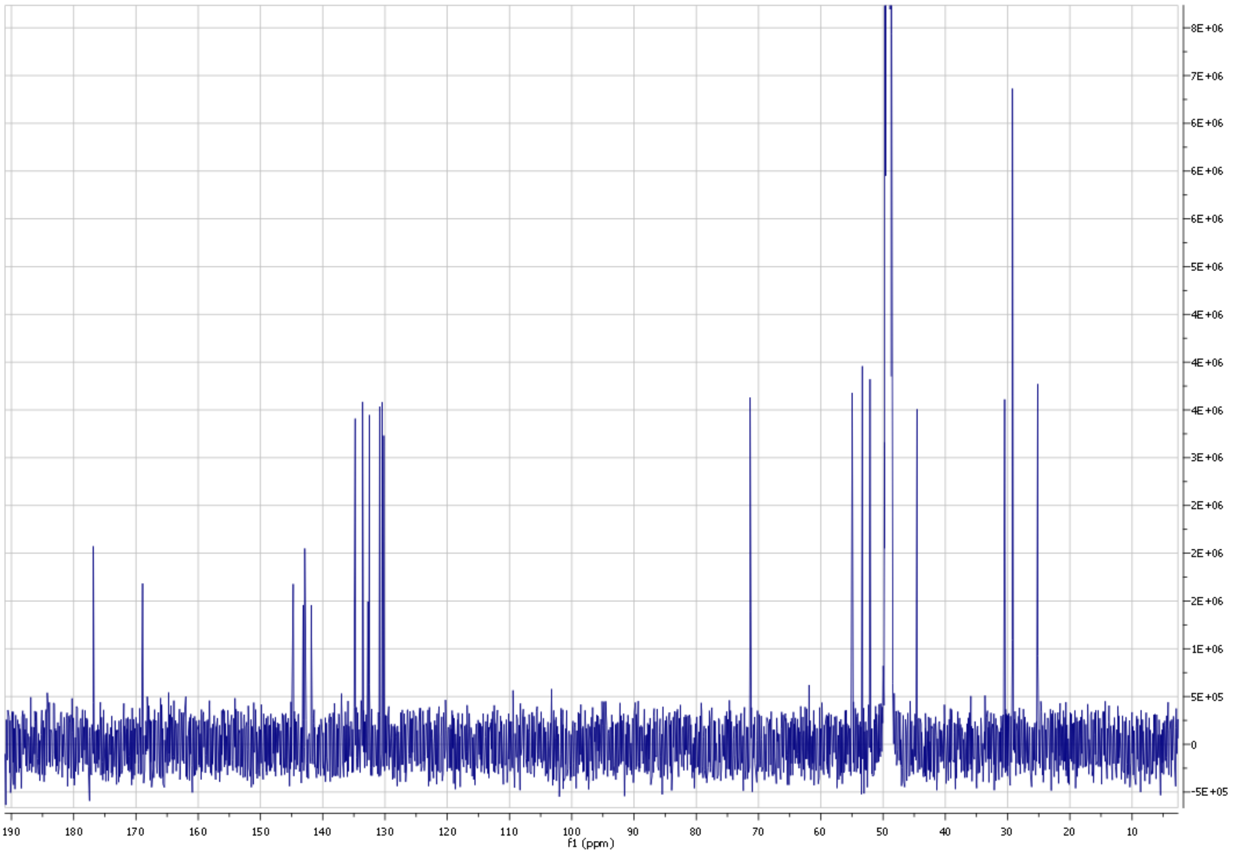

Figure S4. DEPT of compound streptophenazine I (**1**) in MeOD-d_4.

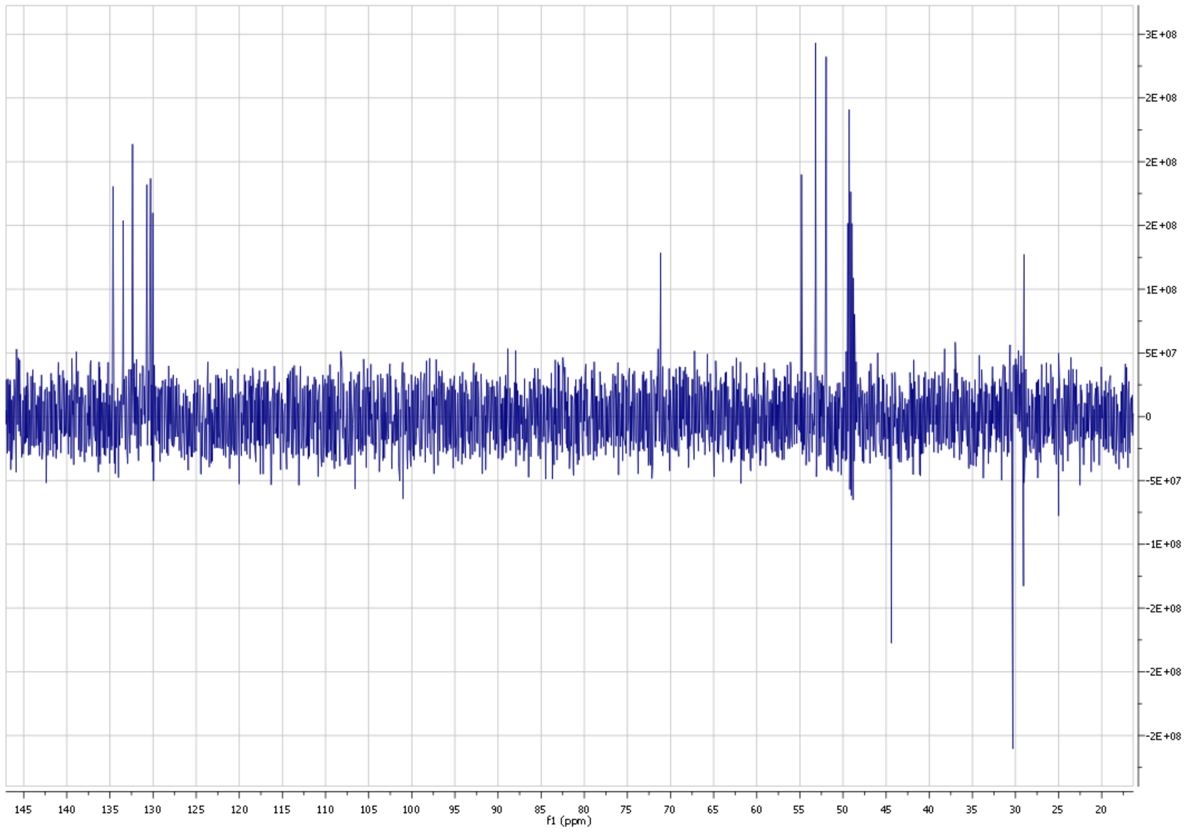

Figure S5. HSQC of compound streptophenazine I (**1**) in MeOD-d_4.

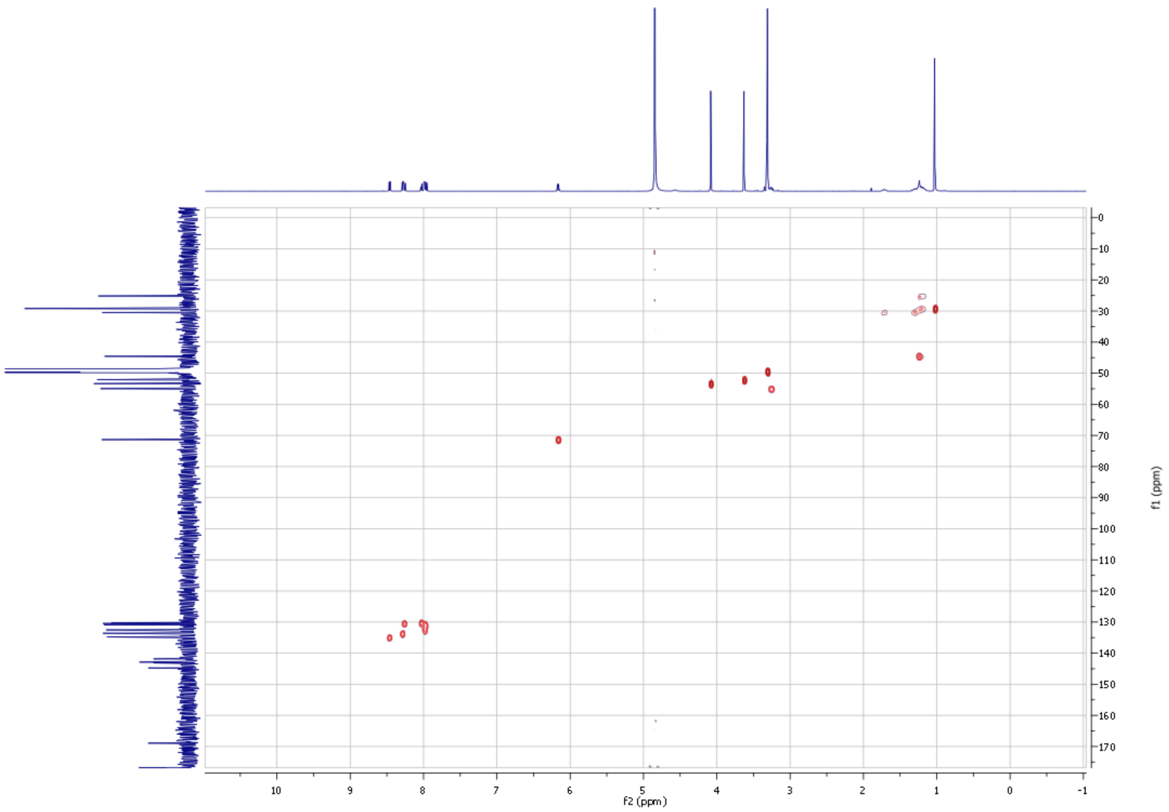

Figure S6. HMBC of compound streptophenazine I (**1**) in MeOD-d_4.

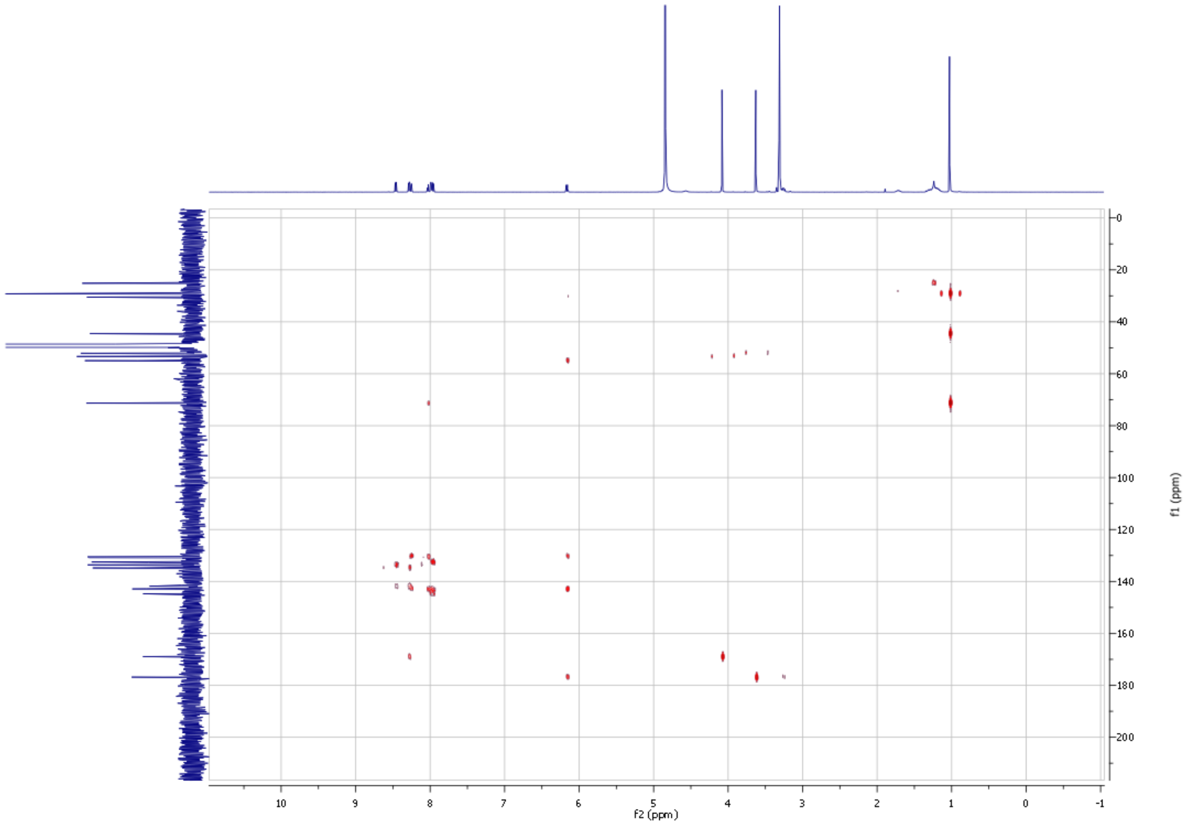

Figure S7. ^{1}H NMR of compound streptophenazine J (**2**) in MeOD-d_4.

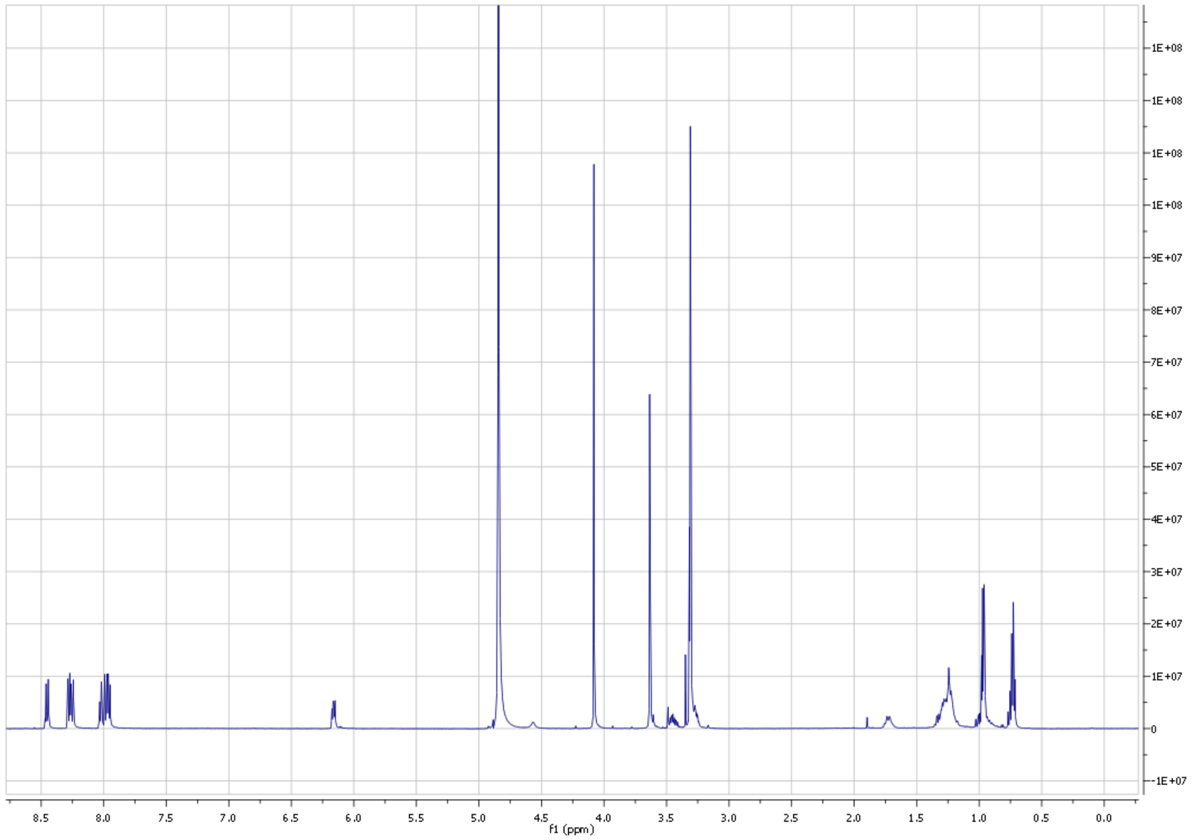

Figure S8. COSY of compound streptophenazine J (**2**) in MeOD-d_4.

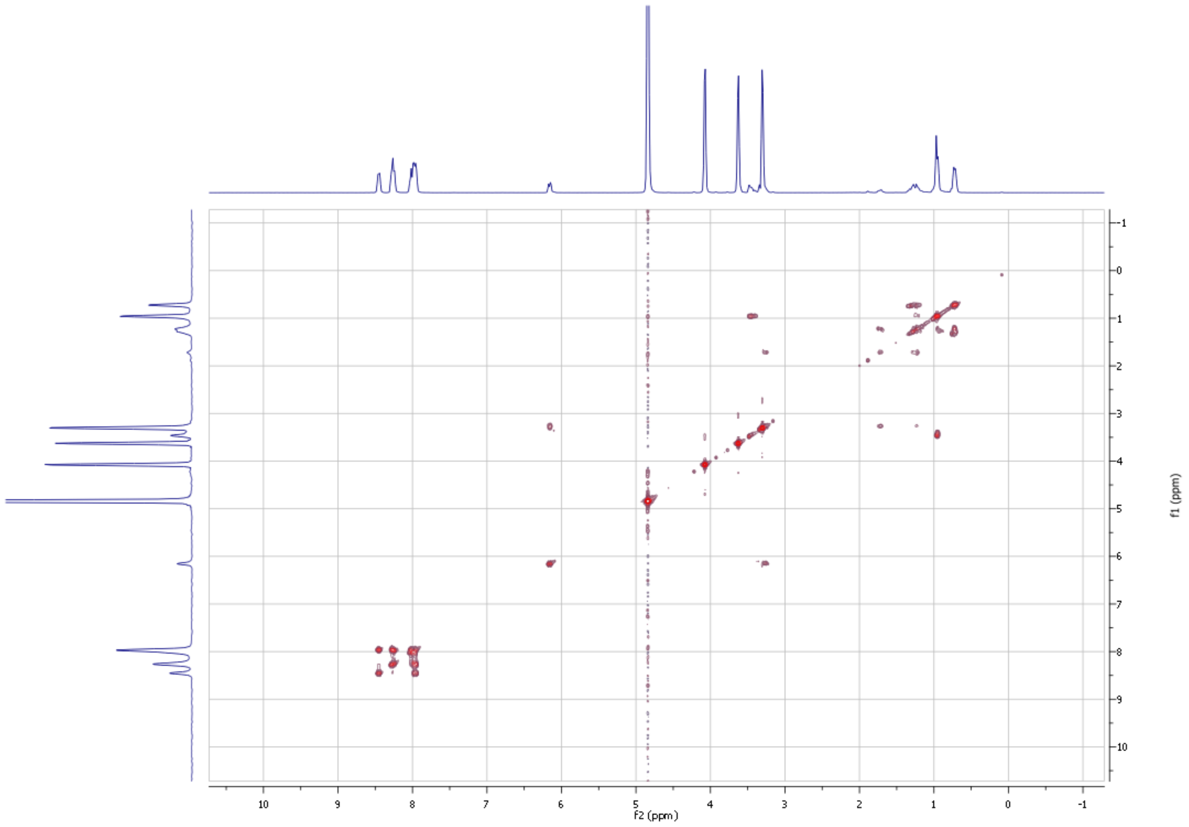

Figure S9. ^{13}C NMR of compound streptophenazine J (**2**) in MeOD-d_4.

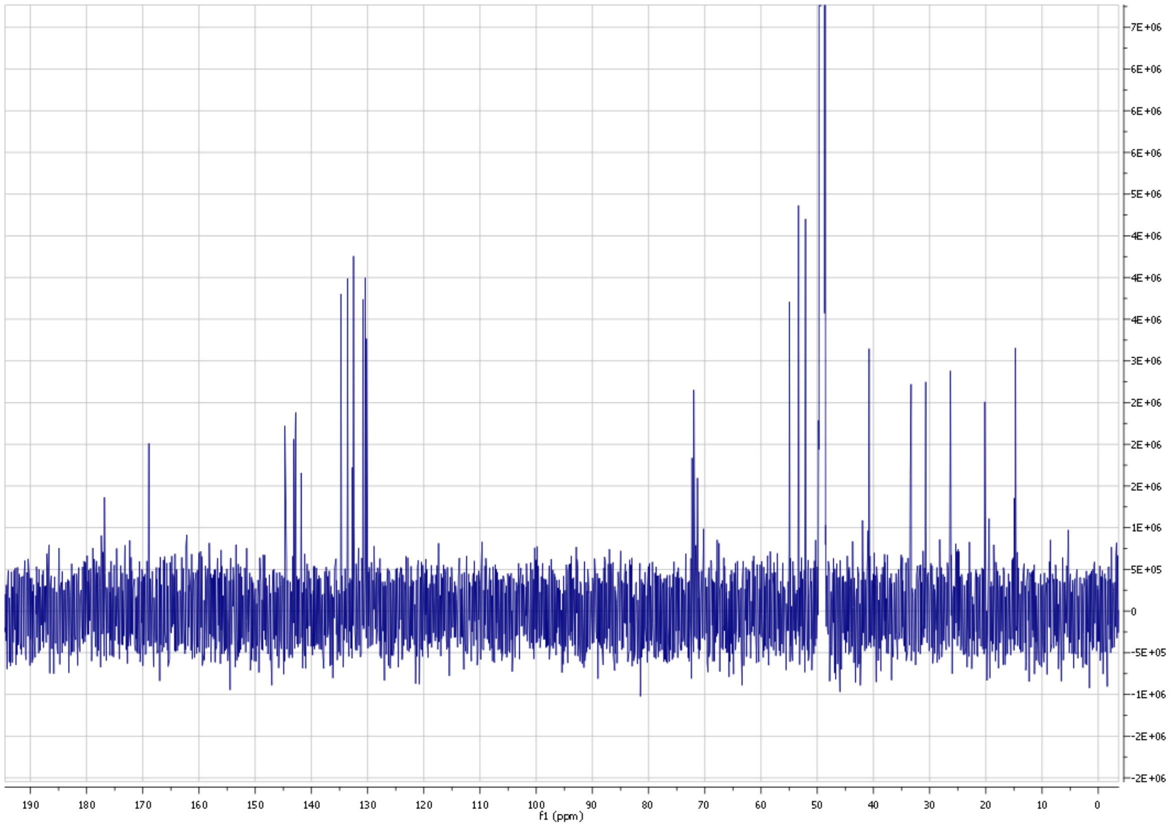

Figure S10. DEPT of compound streptophenazine J (**2**) in MeOD-d_4.

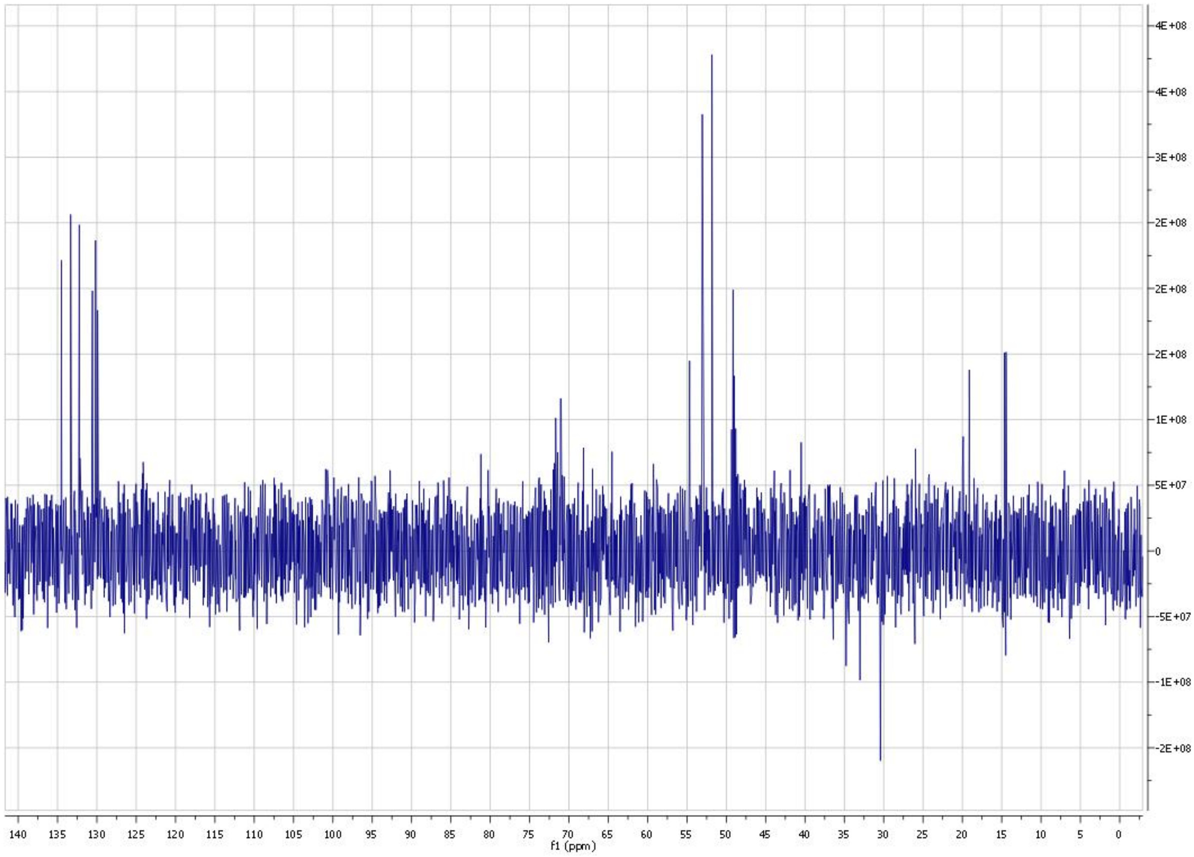

Figure S11. HSQC of compound streptophenazine J (**2**) in MeOD-d_4.

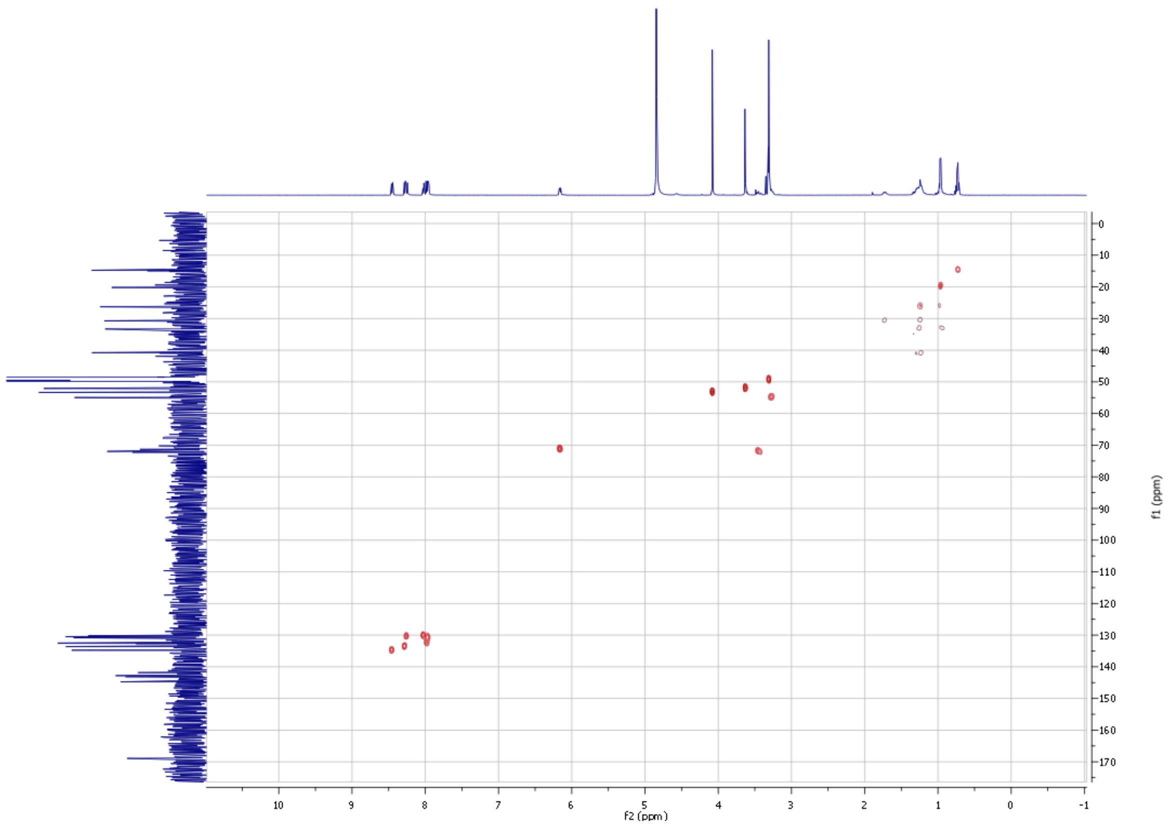

Figure S12. HMBC of compound streptophenazine J (**2**) in MeOD-d_4.

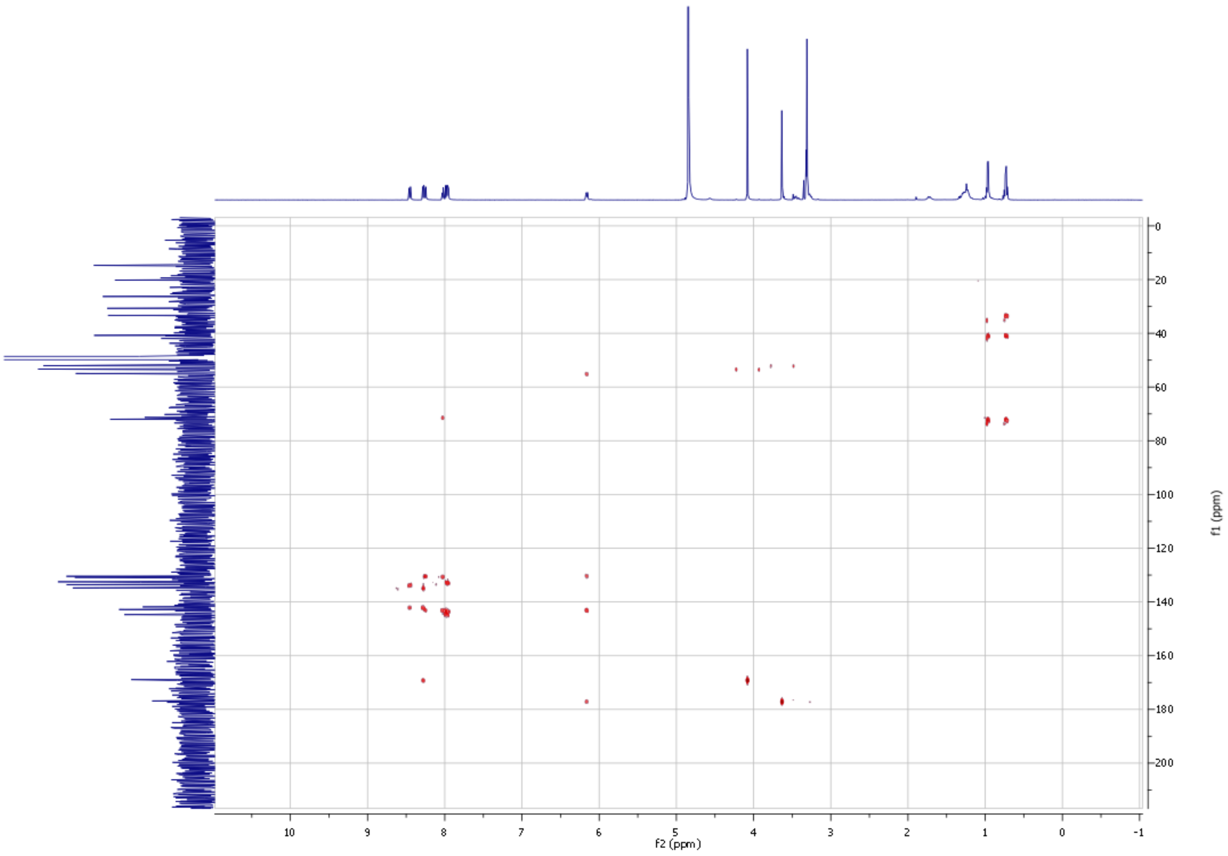

Figure S13. ^{1}H NMR of compound streptophenazine K (**3**) in MeOD-d_4.

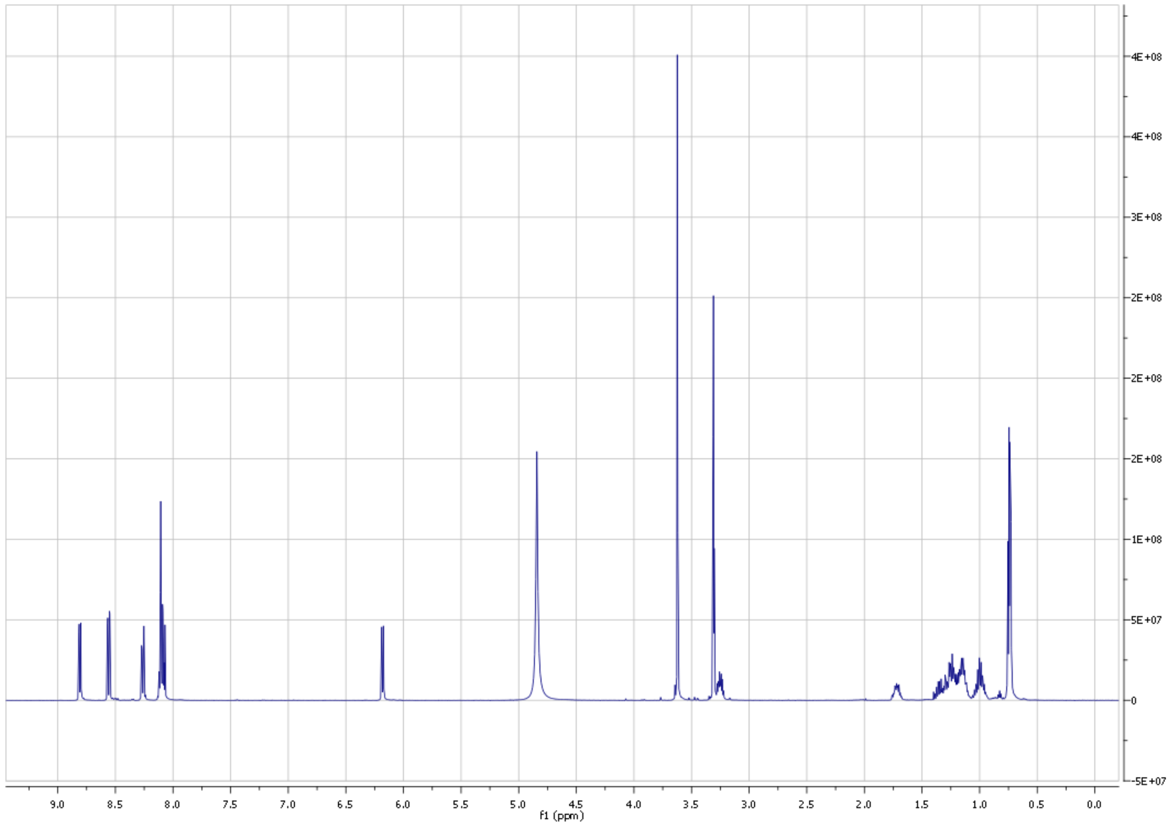

Figure S14. COSY of compound streptophenazine K (**3**) in MeOD-d_4.

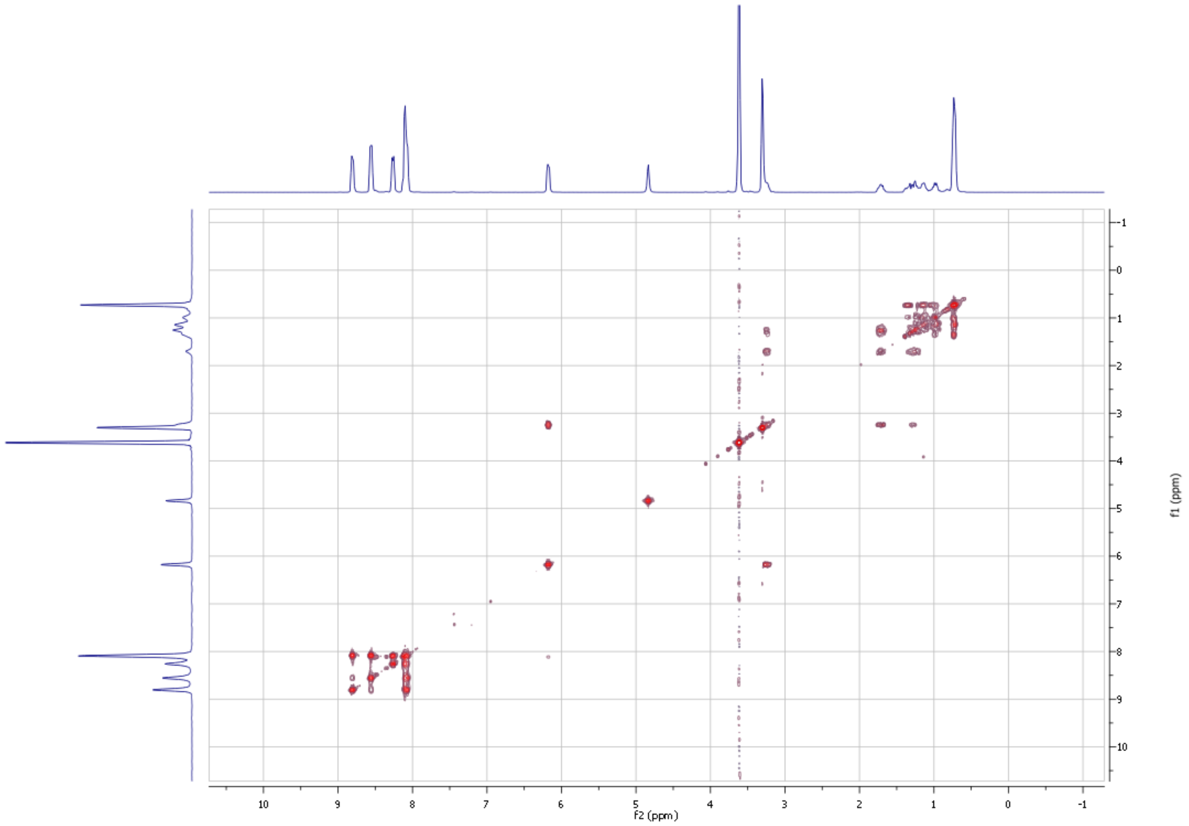

Figure S15. ^{13}C NMR of compound streptophenazine K (**3**) in MeOD-d_4.

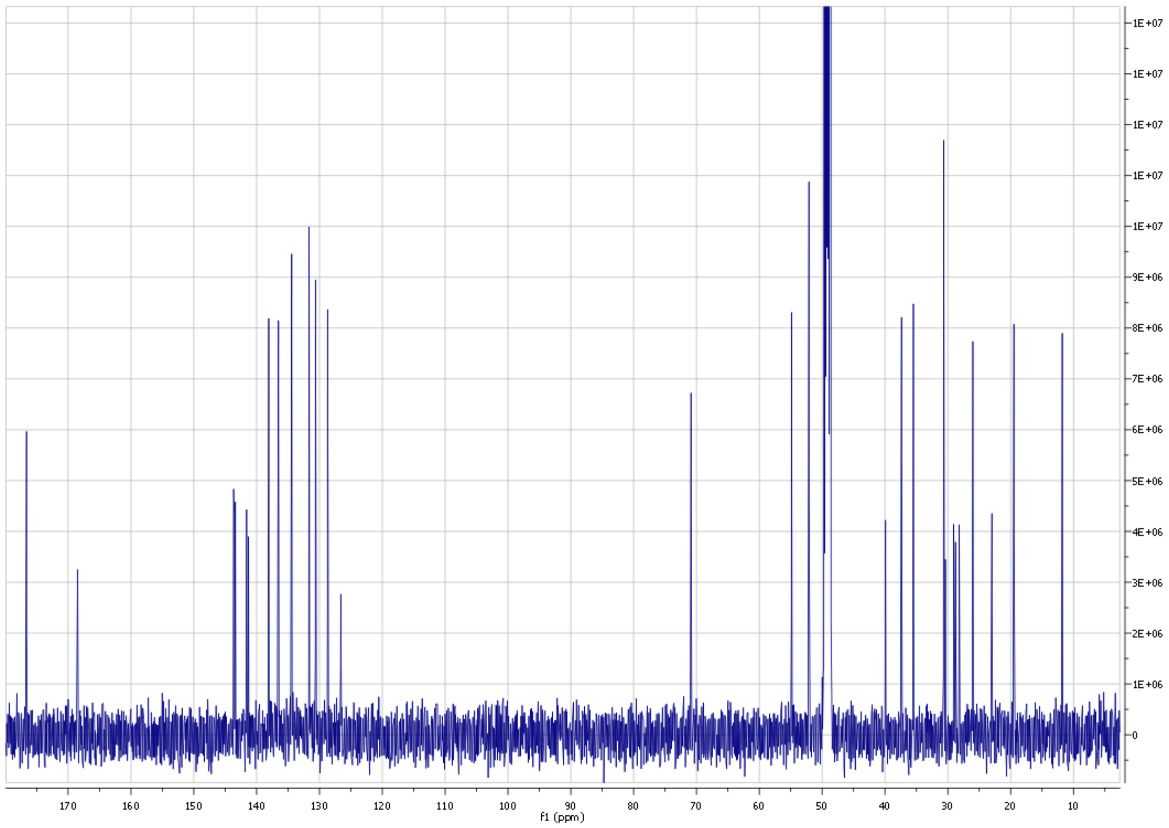

Figure S16. DEPT of compound streptophenazine K (**3**) in MeOD-d_4.

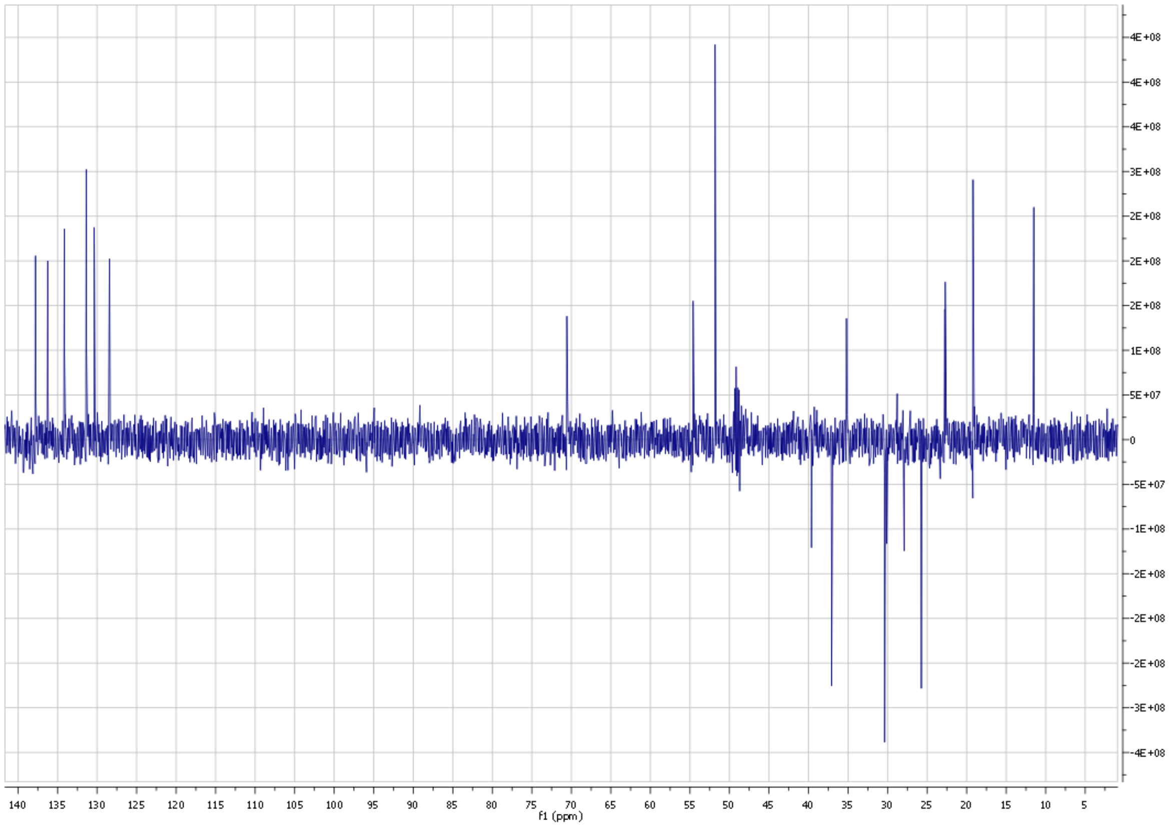

Figure S17. HSQC of compound streptophenazine K (**3**) in MeOD-d_4.

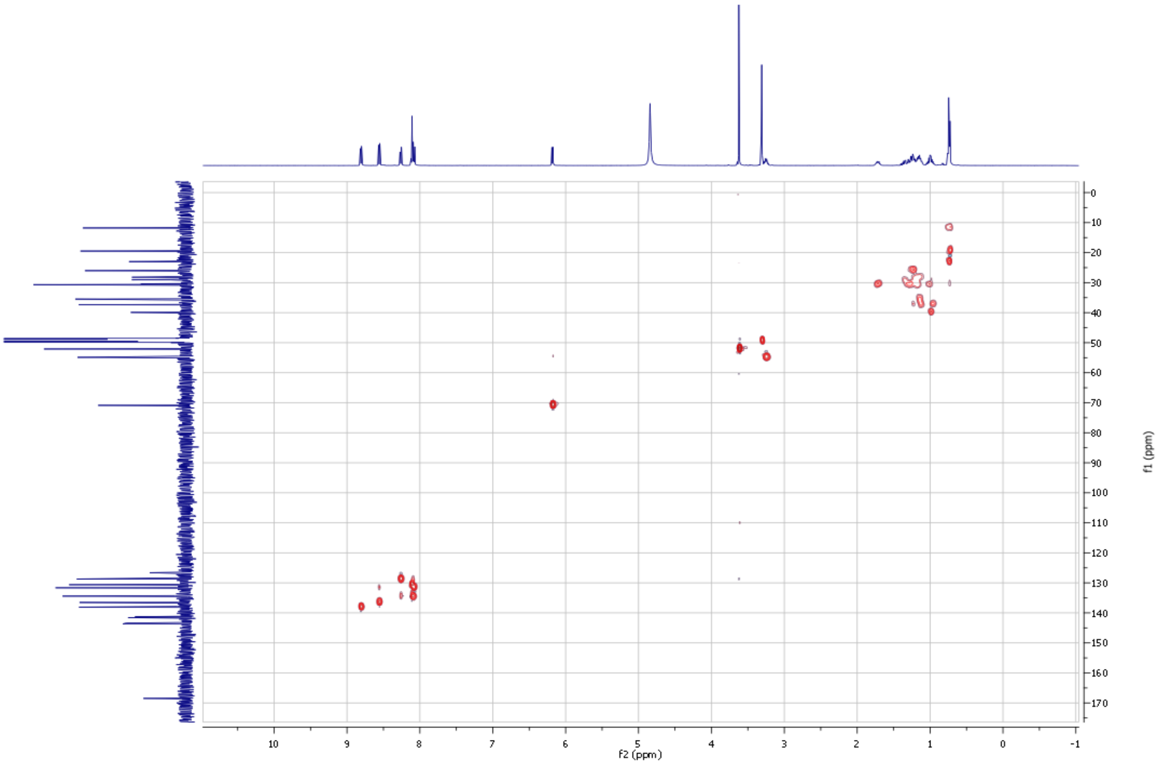

Figure S18. HMBC of compound streptophenazine K (**3**) in MeOD-d_4.

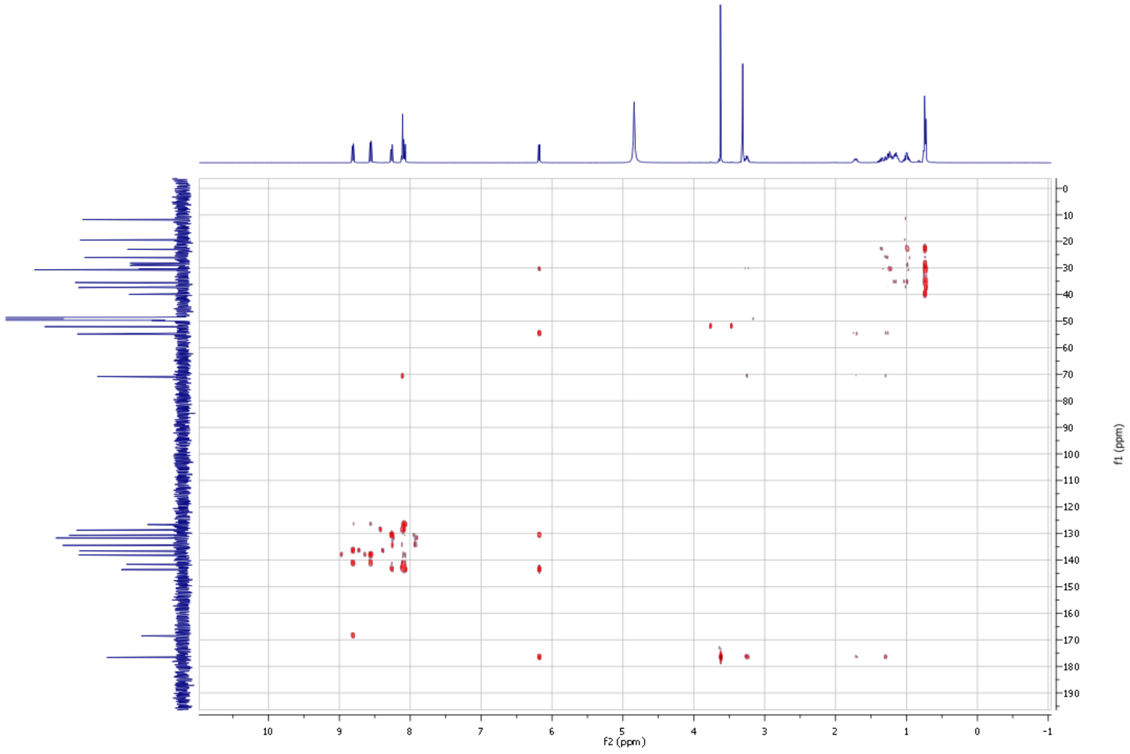

© 2014 by the authors; licensee MDPI, Basel, Switzerland. This article is an open access article distributed under the terms and conditions of the Creative Commons Attribution license (http://creativecommons.org/licenses/by/3.0/).
